# An open comminuted tibia fracture including a 5 cm tibial bone defect in a child: Successful management using a locking compression plate

**DOI:** 10.1016/j.tcr.2023.100944

**Published:** 2023-09-24

**Authors:** Lotje A. Hoogervorst, Bart W. Oudelaar, Demien Broekhuis

**Affiliations:** a Department of Orthopaedics, Leiden University Medical Center, Leiden, the Netherlands; b Department of Biomedical Data Science and Medical Decision Making, Leiden University Medical Center, Leiden, the Netherlands

**Keywords:** Paediatric, Traumatic bone defects, Surgical management, Locking compression plate, Bone grafting

## Abstract

Traumatic bone defects (TBDs), although rare in children, are severe injuries that often represents a challenge for both orthopaedic and trauma surgeons. We present a case of a 6-year-old girl who sustained an open (Gustilo-Anderson type II) comminuted tibia fracture including a ± 5.0 cm distal tibial TBD following a road traffic accident. Open reduction and internal fixation with a 3.5 Locking Compression Plate (LCP) without additional bone grafting was performed, followed by cast immobilization for four months. One and a half years after reconstruction, the patient regained pain-free activity including full-range of motion of her leg and radiographs showed good tibial and fibular alignment, the presence of fracture consolidation and sufficient filling of the TBD. This case report aims to show first evidence of the safety and efficacy of single LCP plating followed by cast immobilization applied in a paediatric patient with a large tibial TBD.

## Introduction

Paediatric traumatic bone defects (TBDs) are rare [1,2]. Treatments of paediatric TBDs are challenging and influenced by multiple factors (e.g. defect size, soft tissue injury and patients' comorbidities). Moreover, there is no consensus in the literature on how to treat paediatric TBDs and which treatment strategy will result in the best outcome.

We report our experience of a paediatric open comminuted tibia fracture including a ± 5.0 cm tibial TBD treated with a Locking Compression Plate (LCP) without additional bone grafting. To the best of our knowledge, this treatment strategy has not been described before.

## Case report

**Fig. 1:**
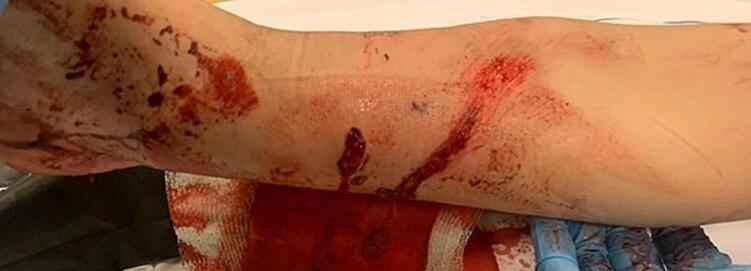
Picture of patients' condition on presentation in the Emergency Department, showing two slightly contaminated puncture wounds on the medial side of the distal tibia of approximately 2- and 3-cm in length (Gustilo-Anderson classification type II).

**Fig. 2:**
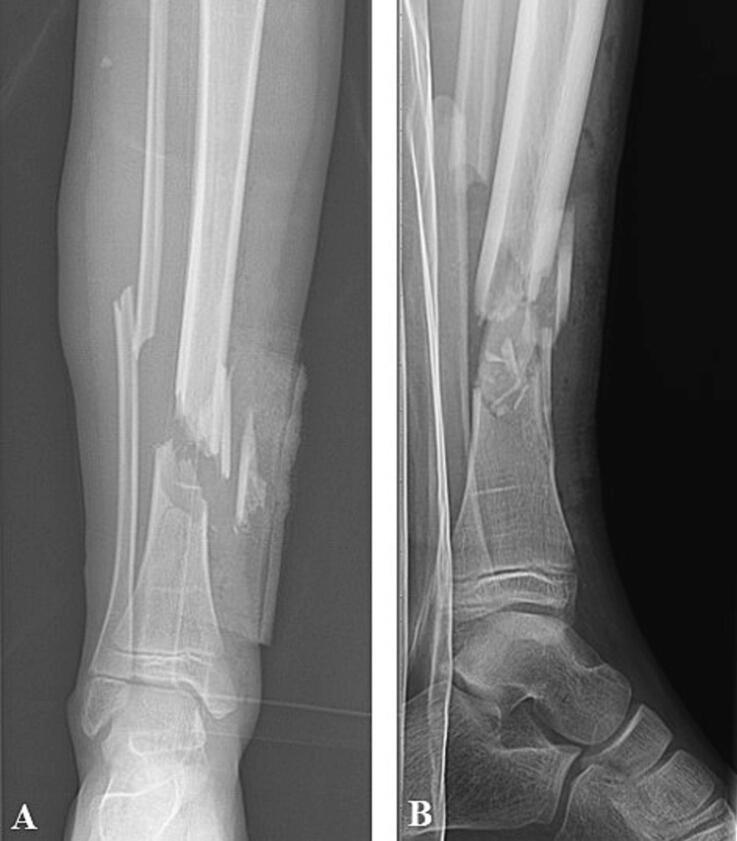
Trauma X-ray, anterior-posterior (**A**) and lateral (**B**) views, showing a right comminuted tibia fracture including a ± 5.0 cm tibial TBD and a dorsal-lateral translated distal fibula fracture.

A previously healthy 6-year-old girl was transferred to a level 1 trauma-centre after she was hit by a scooter. On arrival, the patient was in considerable pain but conscious, oriented and hemodynamically stable. The right lower leg showed a valgus deformity and two slightly contaminated puncture wounds, Gustilo-Anderson type II, on the medial side of the distal tibia (Fig. 1). The foot was neurovascularly intact. X-rays of the leg revealed a comminuted fracture of the distal tibia including a ± 5.0 cm tibial TBD and a dorsal-lateral translated distal fibula fracture (Fig. 2). The girl received 1 g cefazolin intravenously and was taken to theatre for surgical treatment.

**Fig. 3:**
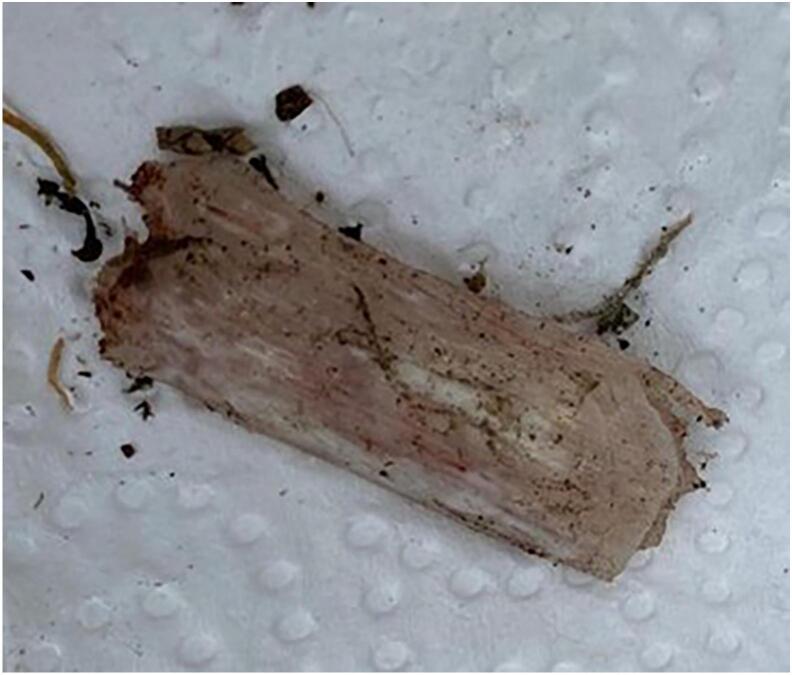
Picture of the missing tibial bone fragment which was later found on the street.

**Fig. 4:**
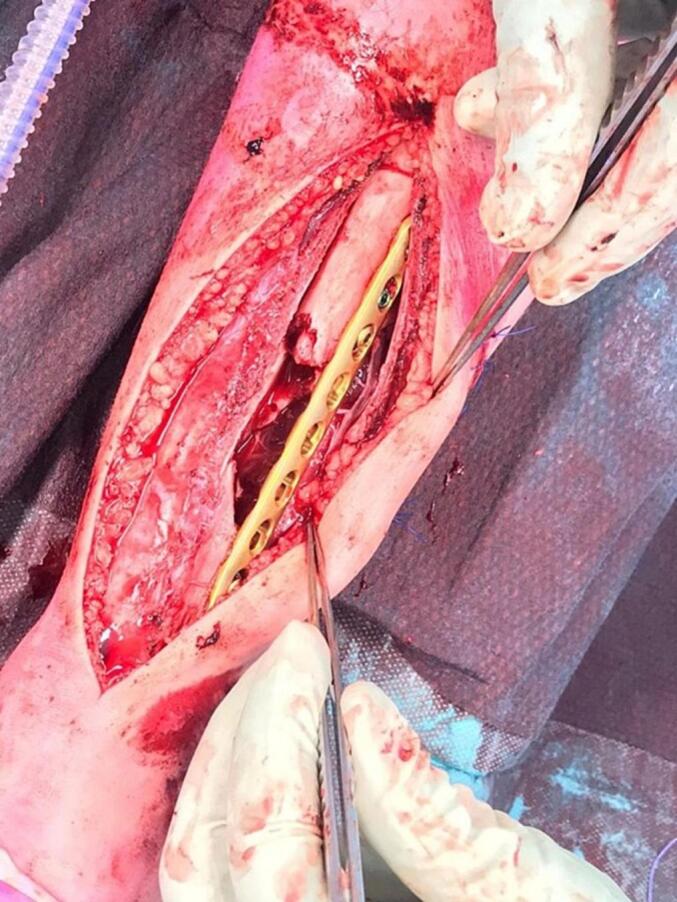
Intraoperative picture showing the large tibial TBD which remained after fixation of the lateral fragment, which was subsequently bridged by the 3.5 LCP.

**Fig. 5:**
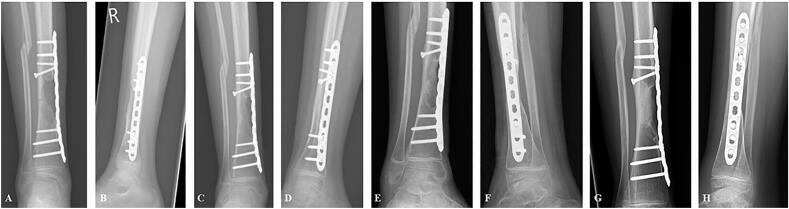
Postoperative X-rays showing increased callus formation and hypertrophy at the fracture site during follow-up; 1 month (**A, B**); 3 months (**C, D**); 6 months (**E, F**); 12 months (**G, H**).

First, the two wounds were debrided sharply. Gross contamination was removed. Second, using an anteromedial approach, the TBD and fragments were explored. A significant portion of the posterior periosteum remained intact, but several bone fragments were disengaged. Despite having found the majority of the fragments, there was still a large piece of tibia missing, which was found on the street (Fig. 3). Third, the largest lateral fragment, which was medially displaced, was reduced and fixed with a lag-screw to create a bone bridge between the proximal and distal tibia. The second largest fragment could, due to deformity, not be restored and was therefore removed. A 3.5 LCP was used to bridge the large tibial TBD (Fig. 4) which was initially fixated with a cortical-screw on each side of the fracture. After correct alignment was verified using fluoroscopy, the LCP was further fixated with: 1) two locking-screws on each side of the fracture; 2) six cortices proximally; and 3) five cortices distally. Intra-operative X-rays were taken for verification of the correct placement of osteosynthesis material. During the index procedure and thereafter, no bone grafting was performed for the following reasons: 1) to reduce the risk of infection of the bone graft, and 2) enough bone formation was observed during follow-up (Fig. 5).

Postoperatively, antibiotic prophylaxis (cefazolin 4 times 1 g in 24 h) was prescribed and a lower leg circular plaster cast was applied to protect the limb. The patient was discharged four days post-operatively with instructions not to weight bear for at least six weeks.

The surgical wound healed without complications. Callus formation was observed three months post-operatively (Fig. 5) which resulted in patients' allowance of flat-foot touchdown weight bearing in a walking cast. Callus formation progressed leading to the allowance of weight bearing as tolerated in a Sarmiento brace four months post-operatively. Nowadays, 1.5 years post-operative, the girl returned her daily activities and progressive radiographic healing was seen (Fig. 5).

## Discussion

Only a small amount of cases describing paediatric TBDs have been reported [[1], [2], [3], [4]], of which the largest series included 27 patients with a mean age of 11.4 years (range:3–16 years) old [3]. Of these patients, six had comparable injury mechanisms (road traffic accidents) and similar sizes of TBD (2-5 cm versus *±* 5.0 cm in our report) and two patients had also Gustilo-Anderson type II injuries. Nevertheless, it could be argued that there was no fully segmental TBD in our patient, due to the created bone bridge. Whether bone bridges were used in the aforementioned cohort is unknown. Two patients were treated with induced membrane, two with induced membrane and intertibiofibular graft (ITFG), one with autograft and ITFG, one with autograft solely and one with bone transport solely. Bone union (not further defined) occurred within a mean of 10 months, which is slightly slower when compared with our case (between 3- and 6-months, depending on the definition used). Complications were often observed in the cohort: infection (five patients), limited range of knee motion (one patient) and an ankle equinus (one patient). Contrarily, our patient recovered without any complications.

Bone loss can be divided into two types: “true” and “in situ” [2,5,6]. “True” bone loss can be caused by extrusion of fragments during trauma [2,5]. Large avascular bony fragments are called “in situ” bone loss [6]. Some authors believe that both types act comparably “it will not heal within 6 months (i.e. nonunion)” [2,6] and that >2 cm bone defects requires additional bone grafting [5]. Surprisingly, our case demonstrated that even a *±* 5.0 cm TBD treated with a LCP solely result in good fracture healing. Good prognostic factors in our case were: 1) the ability to immediately restore the majority of the TBD by creating a bone bridge, and 2) the tibial periosteum remained partially intact. The periosteum is e.g. responsible for fracture healing [9]. The paediatric periosteum is unique as it is extremely strong, thick, vascularized, active and less tightly connected to cortical bone when compared with adult periosteum [9]. Hence, intact periosteums can be observed in paediatric TBDs [9]. In our case, in the presence of a bone bridge and partially intact periosteum, enough bone formation was observed to provide sufficient functional strength, precluding the need for additional bone grafting or other interventions.

To our knowledge, no report has been published describing the use of LCPs in paediatric TBDs. LCPs allow biomechanical forces to be transmitted through screw heads which results in less periosteal disruptions and bone damages when compared with external fixators [7], which makes LCPs suitable in situations with poor bone- quality and/or -quantity [8]. Additionally, LCPs permit application of both cortical- and locking-screws to achieve compression at the fracture end if needed [8].

## Conclusion

We described the case of a paediatric tibial TBD with a partial intact periosteum, a bone bridge and an aseptic bone environment. We showed that the use of a single LCP followed by cast immobilization appears to represent a good treatment regime when treating these kind of TBDs.

## Declaration of competing interest

The authors declare that they have no known competing financial interests or personal relationships that could have appeared to influence the work reported in this paper.
